# Supplementary material for: Transposable element-mediated rearrangements are prevalent in human genomes
Source: Nat Commun. 2022 Nov 19;13:7115. doi: 10.1038/s41467-022-34810-8 (PMC9675761; doi:10.1038/s41467-022-34810-8)
Supplement: Supplementary file 1 — Supplementary Information [file 41467_2022_34810_MOESM1_ESM.pdf]

SUPPLEMENTARY INFORMATION

Transposable element-mediated rearrangements are prevalent in human genomes

Parithi Balachandran[1], Isha A. Walawalkar[1], Jacob I. Flores[1], Jacob N. Dayton[1], Peter A. Audano[1] & Christine R. Beck[1,2,3*]

[1]The Jackson Laboratory for Genomic Medicine, Farmington CT 06032.

[2]Department of Genetics and Genome Sciences, University of Connecticut Health Center, Farmington, CT 06030.

[3]Institute for Systems Genomics, University of Connecticut, Storrs, CT 06269.

*email: christine.beck@jax.org

TABLE OF CONTENTS

- **Figures**
  - Supplementary Fig. 1. Different approaches used for merging and filtering SVs using SRS and LRS data.
  - Supplementary Fig. 2. Size distribution of 493 TEMRs identified from three samples.
  - Supplementary Fig. 3. Summary of SV length among TEMRs and non-TEMRs.
  - Supplementary Fig. 4. Different recombination patterns between the two Alu elements from TEMR-HR.
  - Supplementary Fig. 5. Inversions with complexities at the junctions.
  - Supplementary Fig. 6. Inversion with complexities at the junctions.
  - Supplementary Fig. 7. Complex rearrangement (mCNV) mediated by TEs.
  - Supplementary Fig. 8. Complex rearrangements (mCNVs) mediated by TEs.
  - Supplementary Fig. 9. List of four mCNVs identified in this study.
  - Supplementary Fig. 10. TEMR comparison between human and chimpanzee.
  - Supplementary Fig.11. Ideogram displaying 96 LINE-1 TEMRs.
  - Supplementary Fig. 12. Example of a single *Alu* element involved in two separate rearrangements.
  - Supplementary Fig. 13. Downsampling analysis using short-read HTS.
  - Supplementary Fig. 14. Pipeline for identifying non-redundant high-confident SVs using short-read and long-read HTS data.
  - Supplementary Fig. 15. Rank-based merging SVs.
- **Tables**
  - Supplementary Table 1. Different type of homologous TEs involved in TEMRs.
  - Supplementary Table 2. Primers used for PCR and Sanger sequencing.
  - Supplementary Table 3. Assessment of breakpoint accuracy between manual reconstruction and SV callers.
  - Supplementary Table 4. List of 36 *Alu* and 54 LINE-1 elements involved in 493 TEMRs.
  - Supplementary Table 5. Count of *Alu* and LINE-1 subfamilies involved in 493 TEMRs.
  - Supplementary Table 6. Median percent similarity between different TEs, SV types, and mechanisms.
  - Supplementary Table 7. Summary of the correlation analysis.
  - Supplementary Table 8. Total count of Exonic, intronic and proximal SVs.
  - Supplementary Table 9. Tools and packages.
  - Supplementary Table 10. Restriction enzymes, primers and probes used for ddPCR.
- **Supplementary References**

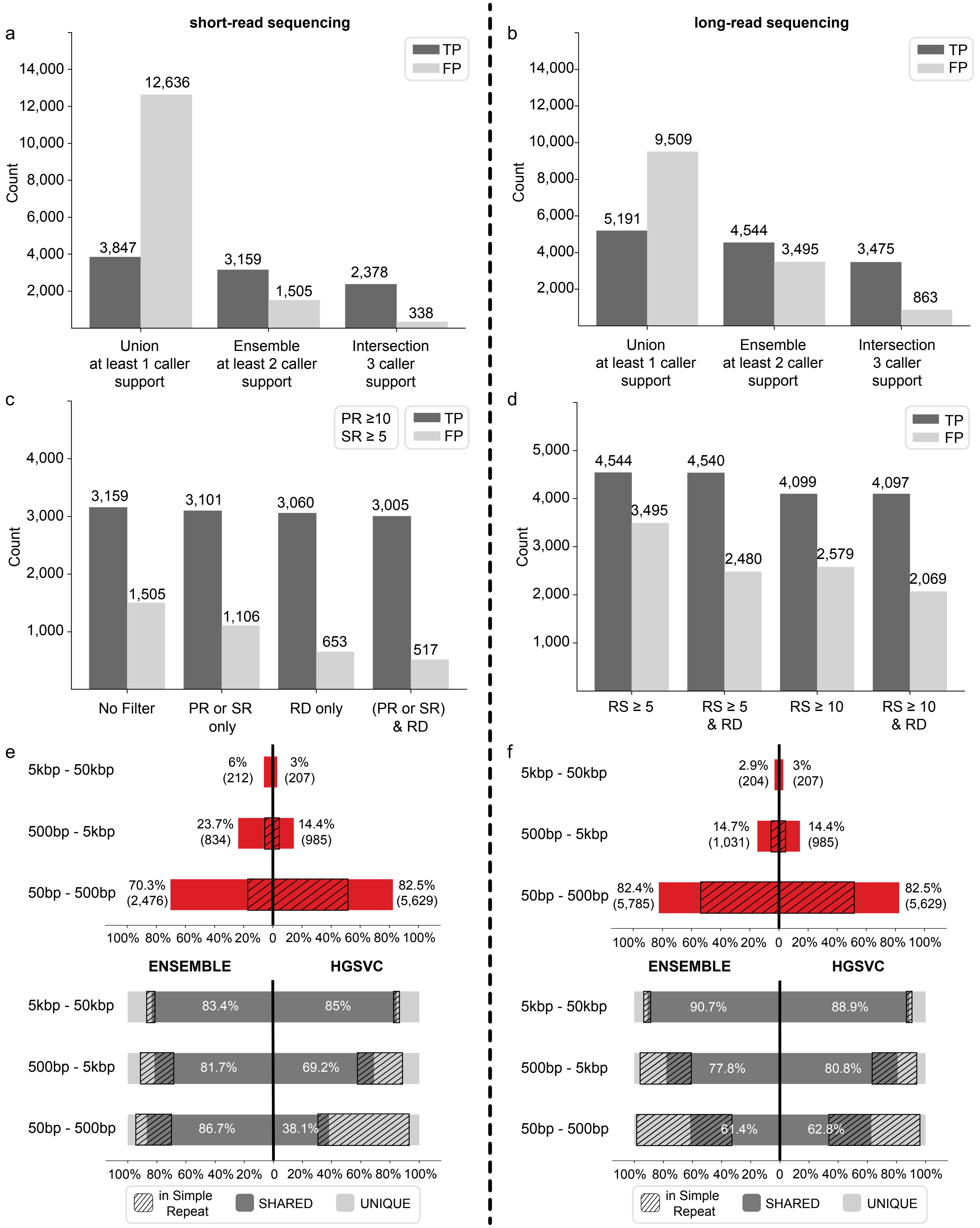

short-read sequencing
long-read sequencing
a
b
c
d
e
f
Count
TP
FP
14,000
12,000
10,000
8,000
6,000
4,000
2,000
3,847
12,636
3,159
1,505
2,378
338
Union at least 1 caller support
Ensemble at least 2 caller support
Intersection 3 caller support
5,191
9,509
4,544
3,495
3,475
863
PR ≥10
SR ≥ 5
3,159
1,505
3,101
1,106
3,060
653
3,005
517
No Filter
PR or SR only
RD only
(PR or SR) & RD
5,000
4,000
3,000
2,000
1,000
4,544
3,495
4,540
2,480
4,099
2,579
4,097
2,069
RS ≥ 5
RS ≥ 5 & RD
RS ≥ 10
RS ≥ 10 & RD
5kbp - 50kbp
500bp - 5kbp
50bp - 500bp
6%
(212)
3%
(207)
23.7%
(834)
14.4%
(985)
70.3%
(2,476)
82.5%
(5,629)
2.9%
(204)
14.7%
(1,031)
82.4%
(5,785)
100% 80% 60% 40% 20% 0 20% 40% 60% 80% 100%
ENSEMBLE
HGSVC
83.4%
85%
81.7%
69.2%
86.7%
38.1%
90.7%
88.9%
77.8%
80.8%
61.4%
62.8%
in Simple Repeat
SHARED
UNIQUE

Supplementary Fig. 1. Different approaches used for merging and filtering SVs using SRS (a, c, and e) and LRS (b, d, and f) data. a & b Merging data from multiple callers. Average true positive SVs (TP: dark gray) compared to the average false positive SVs (FP: light gray) per sample, for methods with at least 1 (Union) or 2 (Ensemble) or 3 (Intersect) caller support for identifying an SV. **c & d** Different parameters (paired-read: PR, split-read: SR, read-depth: RD) used for additional filtering. Average true positive SVs (TP: dark gray) compared to the average false positive SVs (FP: light gray) per sample. **e & f** SVs overlapping (50%) simple repeat regions. Top graph indicates percentage of SVs from ensemble callset and truth set that overlaps simple repeats. Bottom graph indicates the percentage of SVs that are either unique or shared between the ensemble callset and the truth set. The shaded portion indicats the percentage of SVs that overlap (50%) simple repeats.

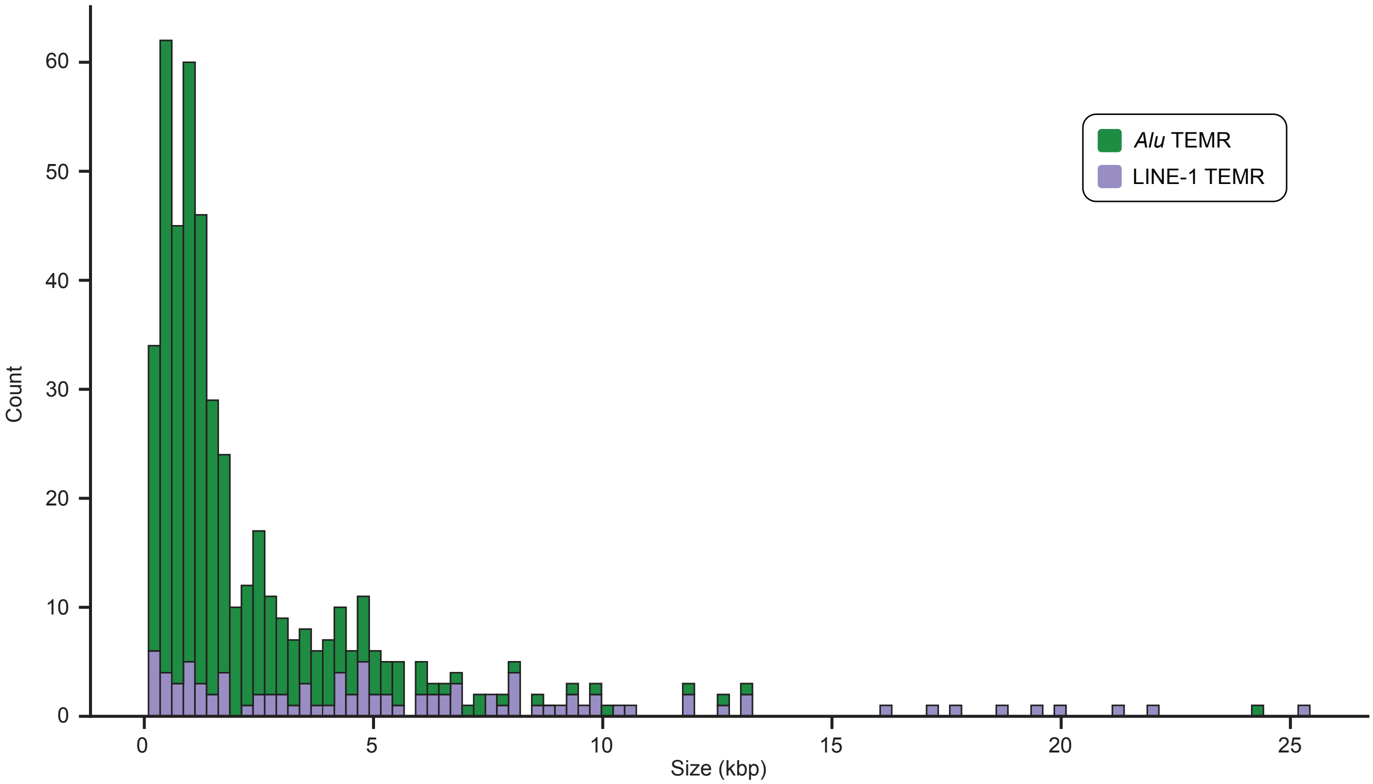

Supplementary Fig. 2. Size distribution of 493 TEMRs identified from three samples. Size distribution of both *Alu* TEMRs (green) and LINE-1 TEMRs (purple) identified in this study.

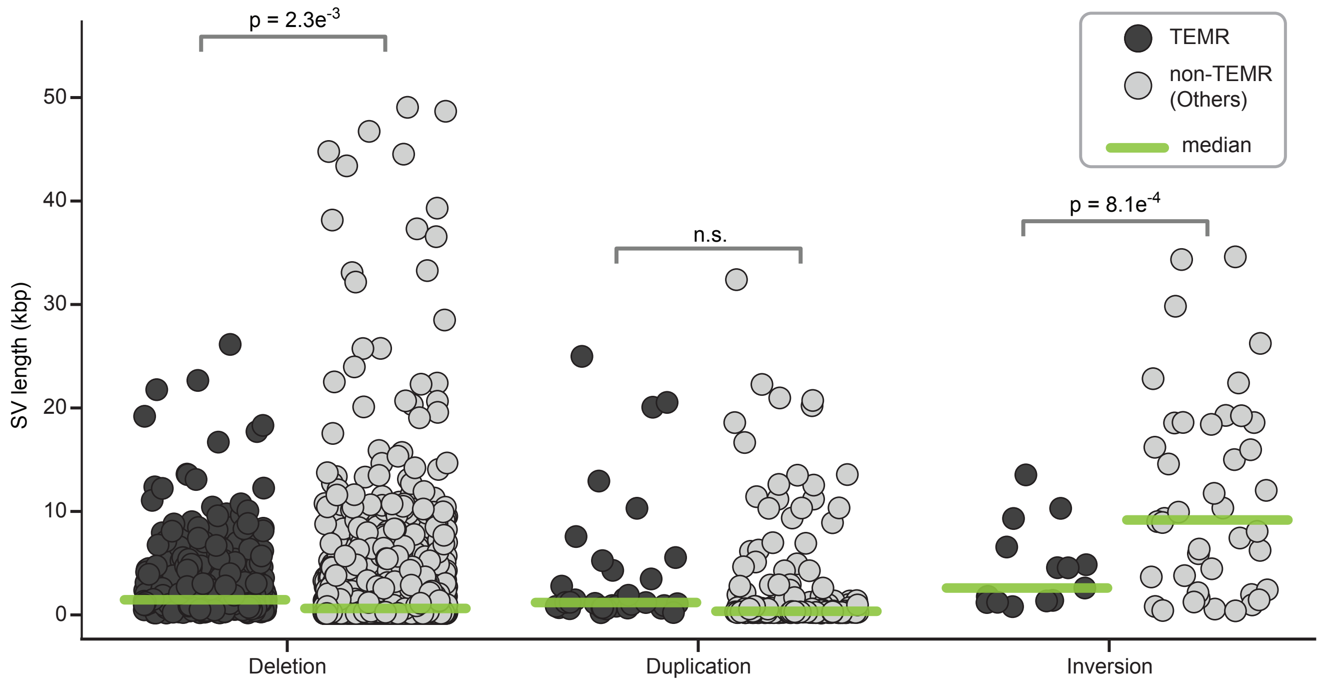

Supplementary Fig. 3. Summary of SV length among TEMRs and non-TEMRs. Summary of SV length among TEMRs and non-TEMRs within deletion (n=445 vs 1,981), duplication (n=33 vs 202), and inversion (n=15 vs 42). Two-sided Welch's t-test was used to calculate the p-value between the two categories.

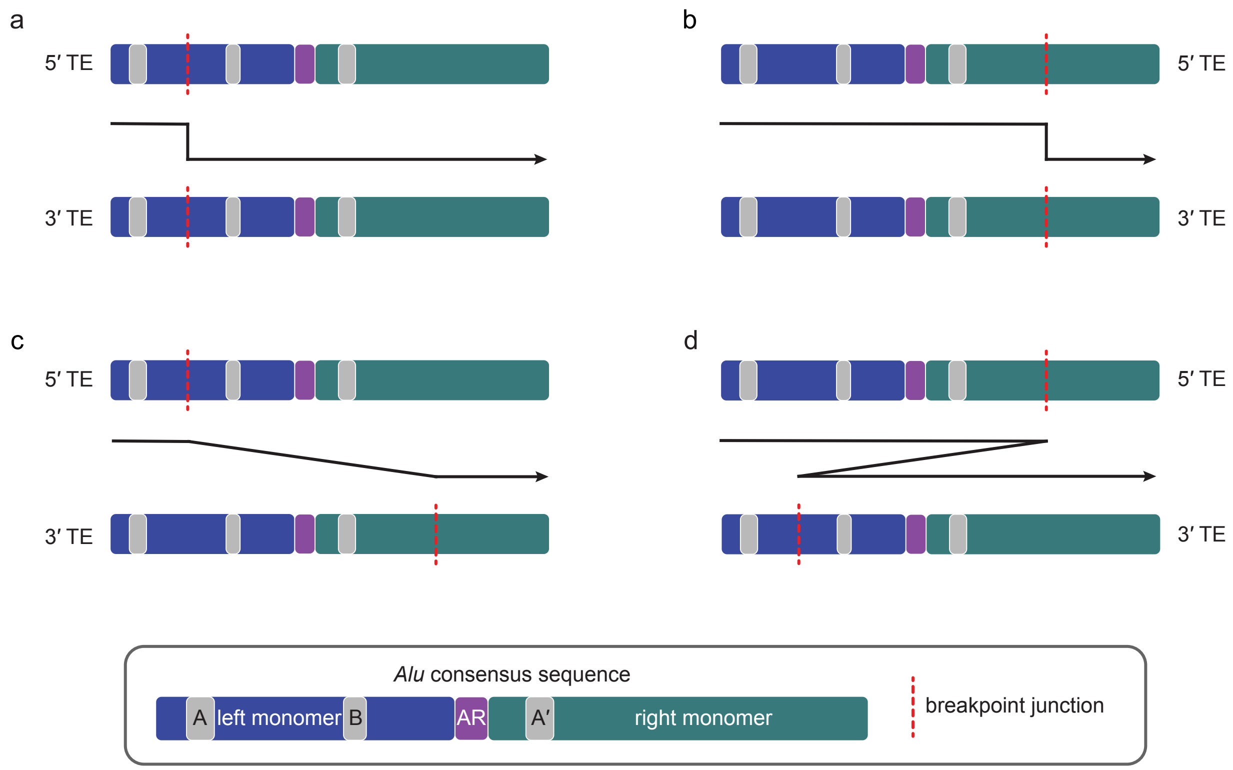

Supplementary Fig. 4. Different recombination patterns between the two *Alu* elements from TEMR-HR. Breakpoint junctions are present in the same monomer (a: left and b: right) of the two *Alu* elements resulting in a ~300 bp chimeric *Alu* recombination product. Breakpoint junctions are present in the opposite monomer of the two *Alu* elements, resulting in a recombination product of either ~150 bp (c) or ~450 bp (d) chimeric *Alu*.

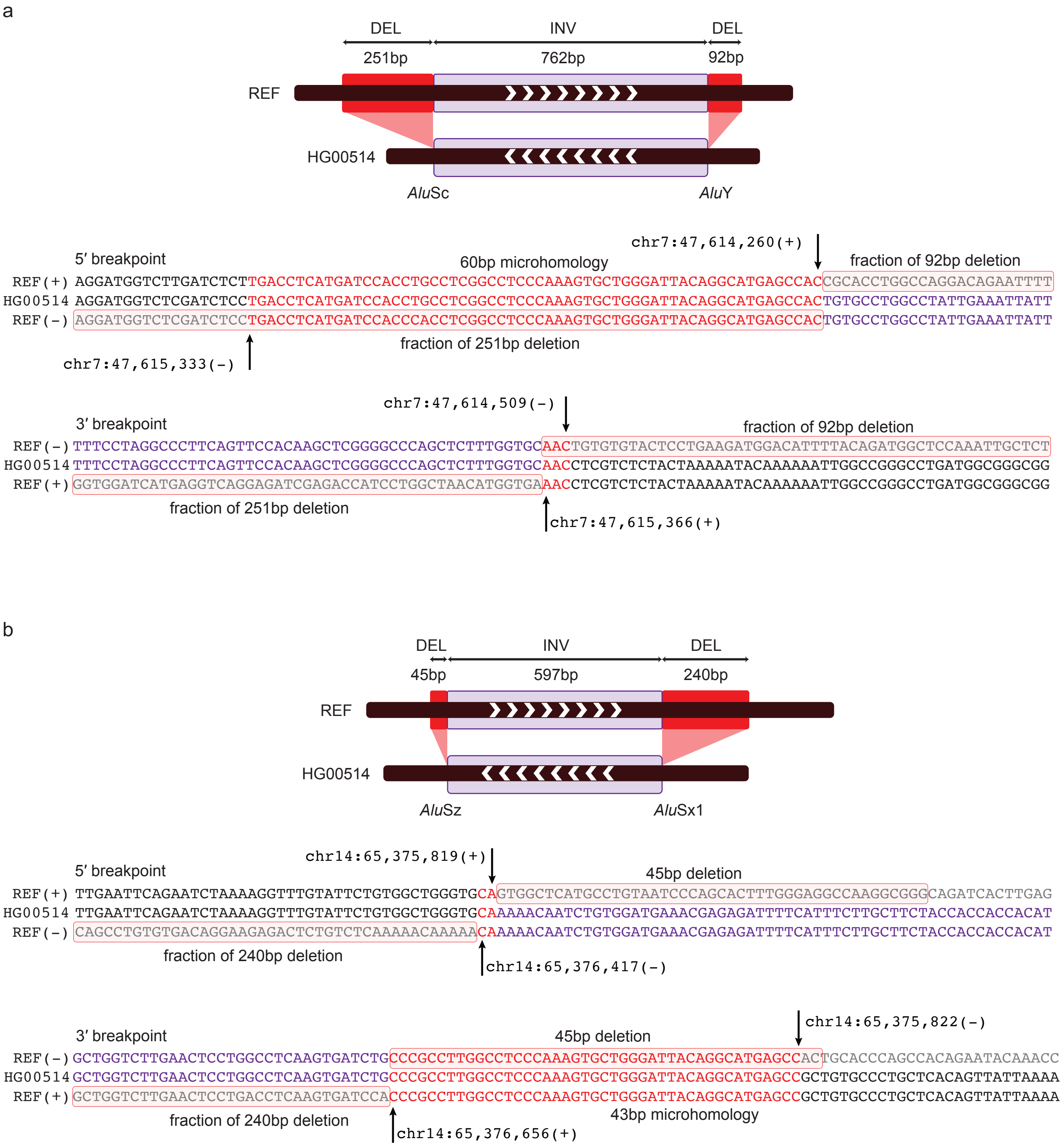

Supplementary Fig. 5. Inversions with complexities at the junctions. Examples of inversion with deletions near the breakpoint junctions. Top panel in a & b: schematic representing the inversion (INV, purple) with deletions (DEL, red) around the breakpoint junction. Bottom panel in a & b: junction reconstruction of the complex inversion (purple font) event with their corresponding microhomologies (red font) and deleted sequence (red box). REF, reference genome (GRCh38).

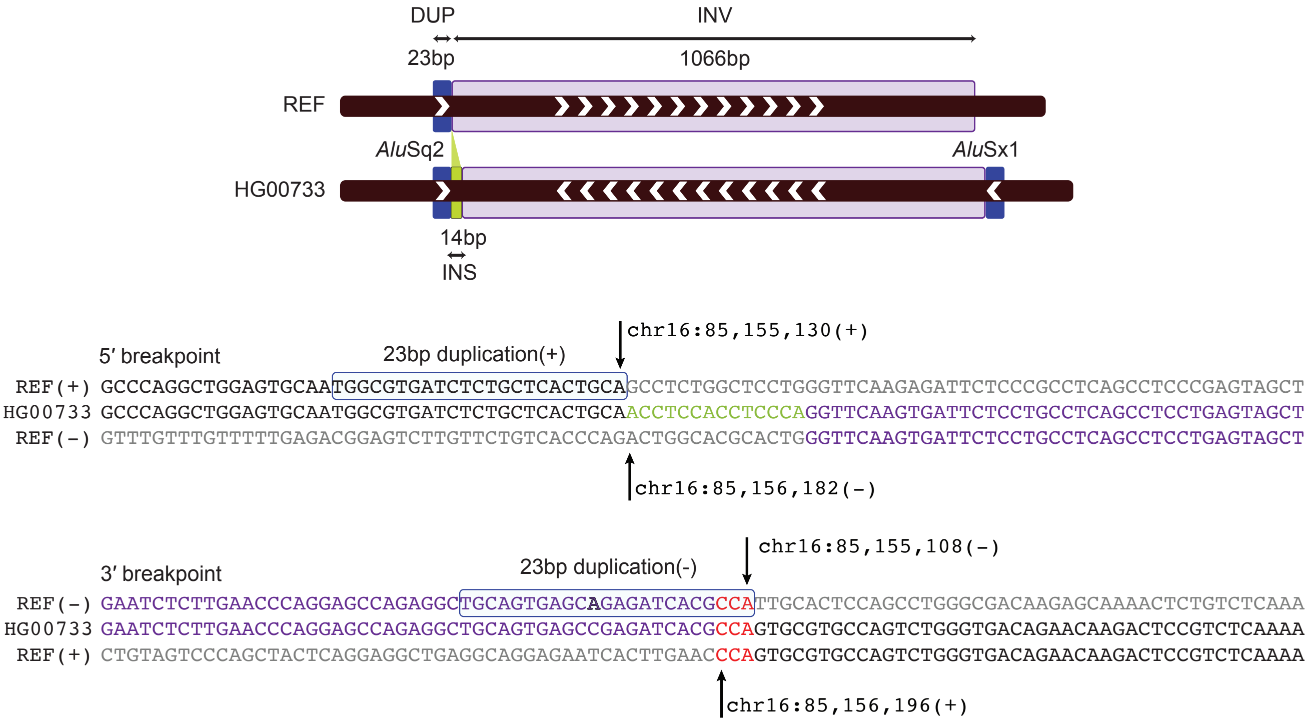

Supplementary Fig. 6. Inversion with complexities at the junctions. Examples of inversion with duplication and insertion near the breakpoint junctions. Top panel: schematic representing the inversion (INV, purple) with duplication (DUP, blue) and insertion (INS, green) around the breakpoint junction. Bottom panel: junction reconstruction of the complex inversion (purple font) event with the corresponding microhomologies (red font), inserted sequence (green font) and duplicated sequence (blue box). REF, reference genome (GRCh38).

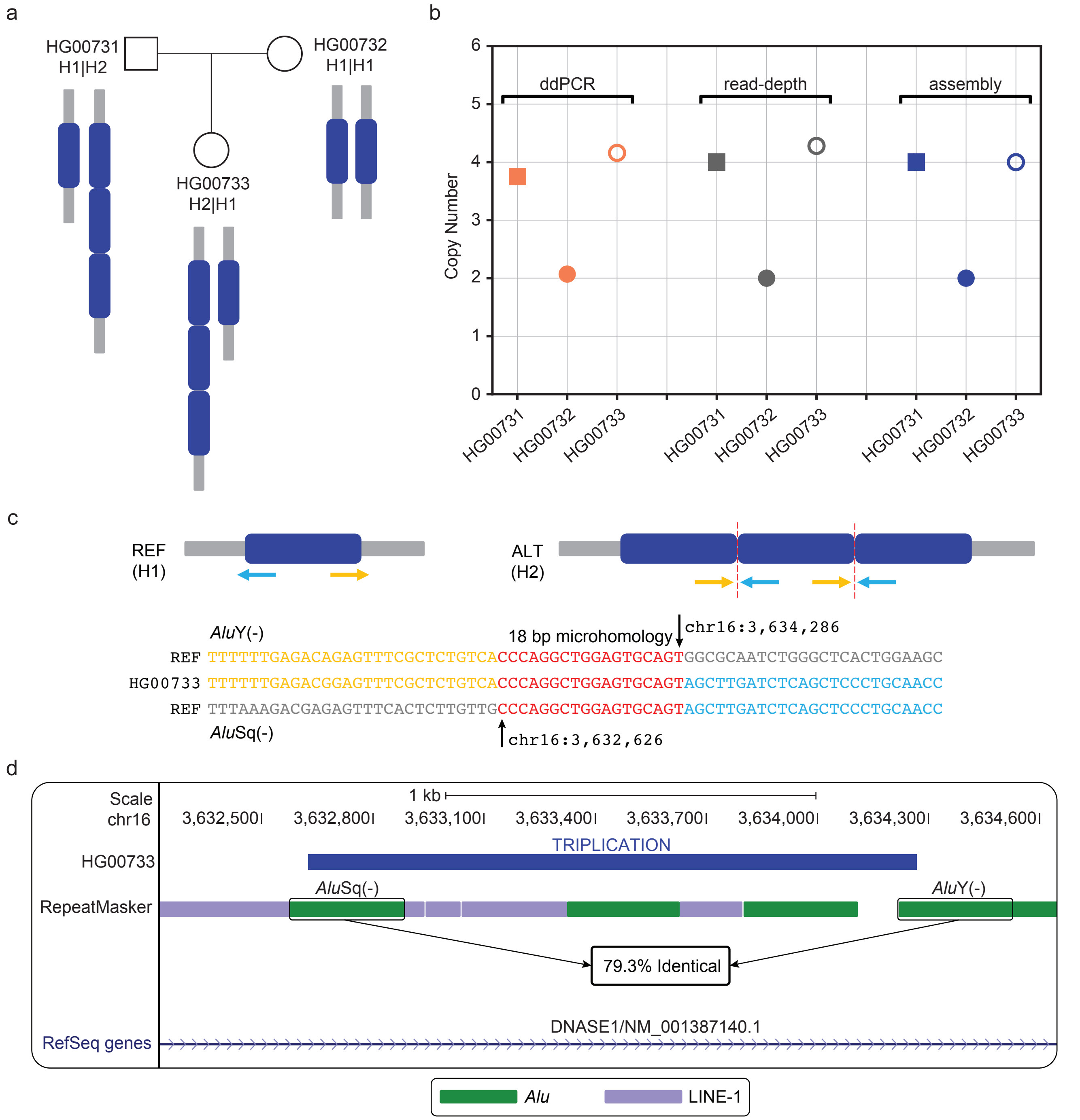

Supplementary Fig. 7. Complex rearrangement (mCNV) mediated by TEs. a Schematic representing the triplication found in the Puerto Rican trio. **b** Copy number status of the individuals containing the triplication identified using ddPCR, read-depth analysis and assembly data. **c** Breakpoint junctions indicating an 18 bp microhomology. **d** UCSC genome browser depicting the triplication between an *Alu*Sq and an *Alu*Y element within intron 1 of *DNASE1*.

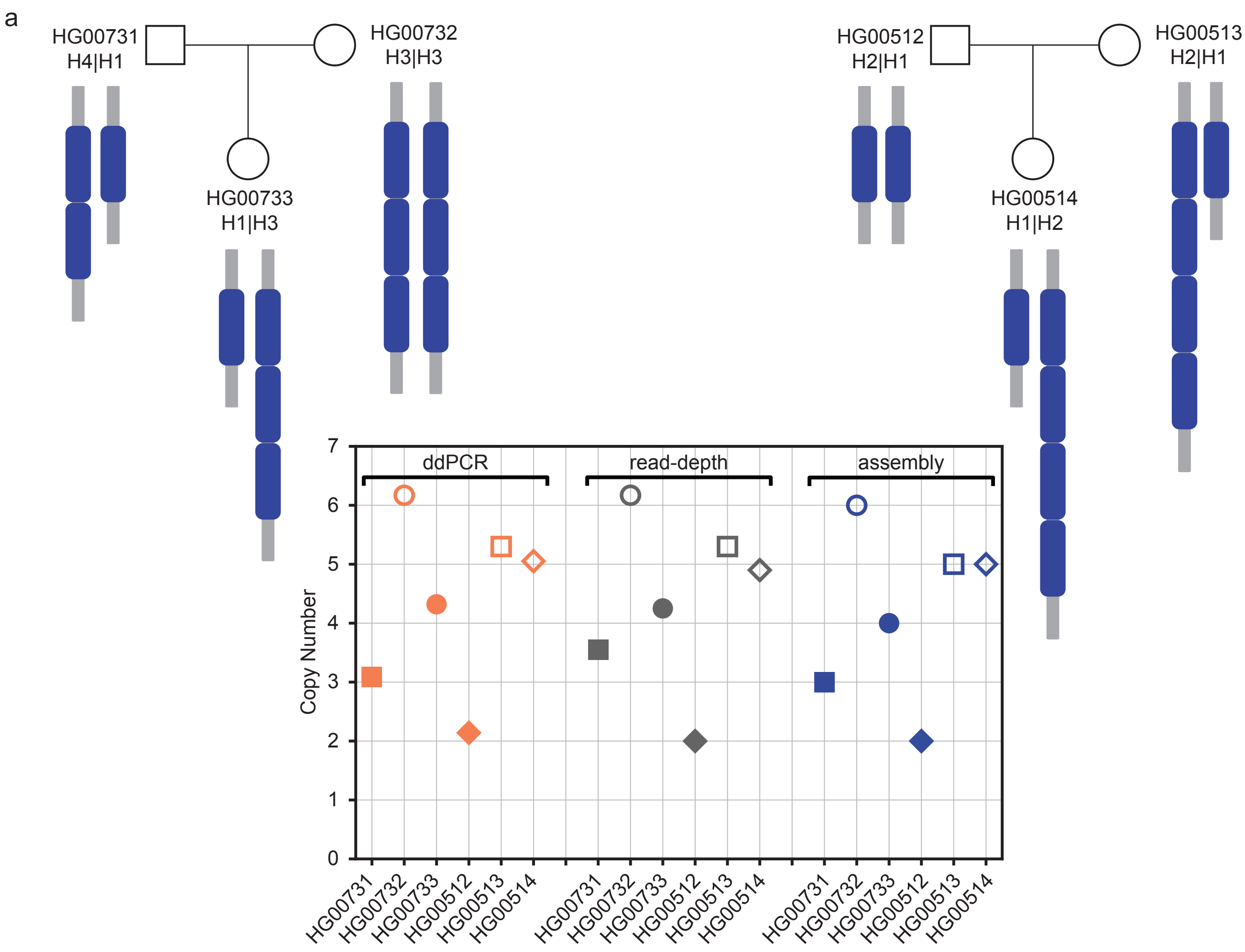

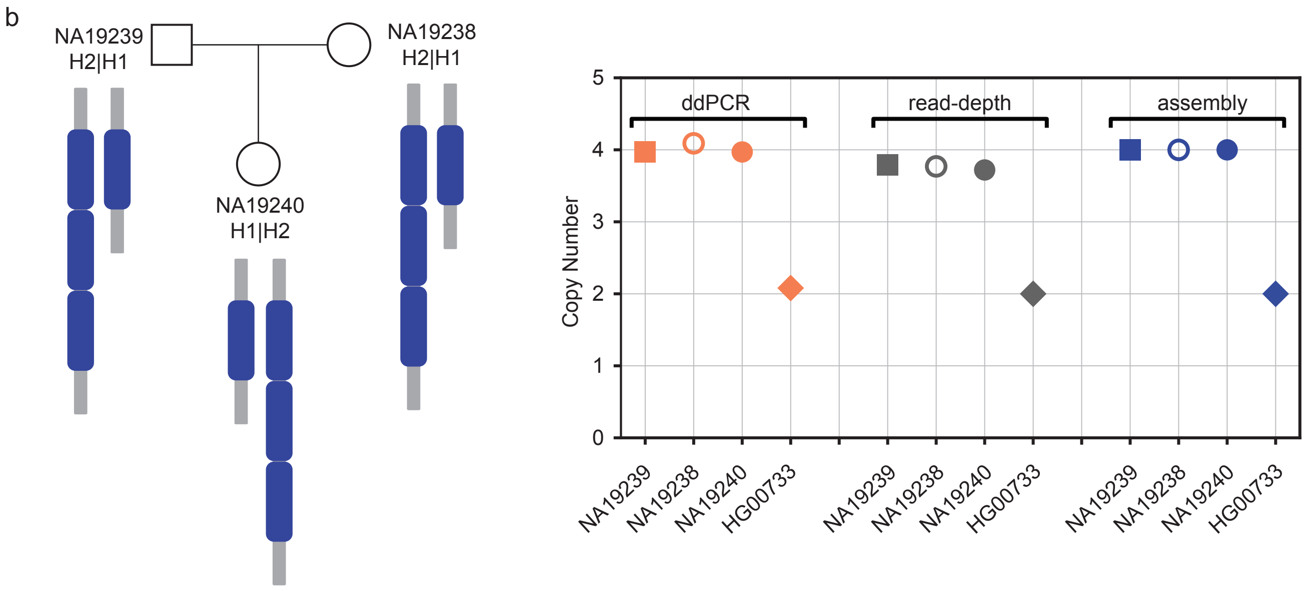

Supplementary Fig. 8. Complex rearrangements (mCNVs) mediated by TEs. a Schematic representing the triplication found in Puerto Rican family and quadruplication found in Han Chinese family. Copy number status of the individuals containing mCNV determined using ddPCR, read-depth analysis and assembly data. **b** Schematic representing the triplication found in the Yoruban Nigerian family. Copy number status of the samples containing triplication determined using ddPCR, read-depth analysis and assembly data.

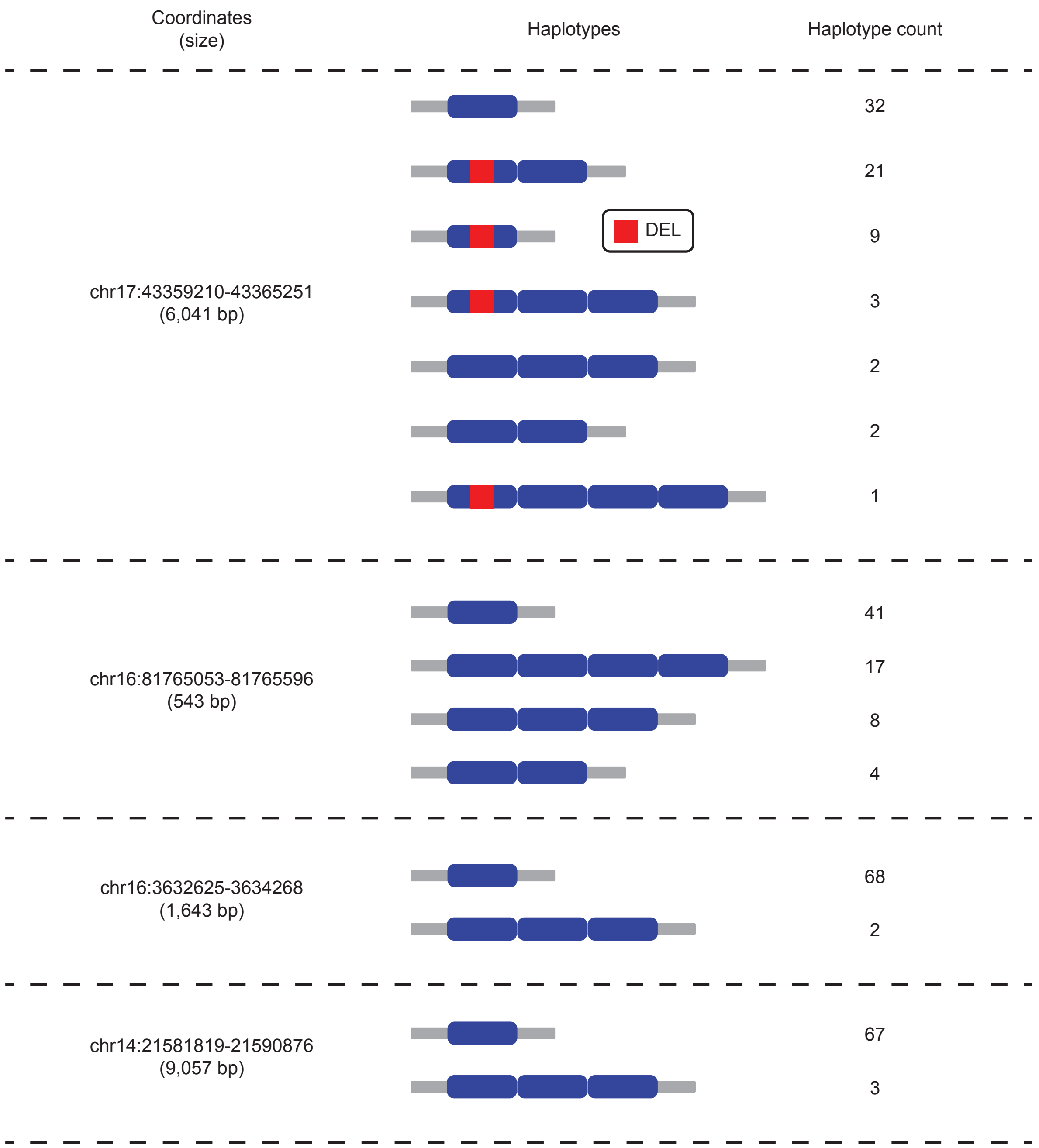

Supplementary Fig. 9. List of four mCNVs identified in this study. *Alu*/*Alu*-mediated amplifications resulting in multiple haplotypes within diverse human population.

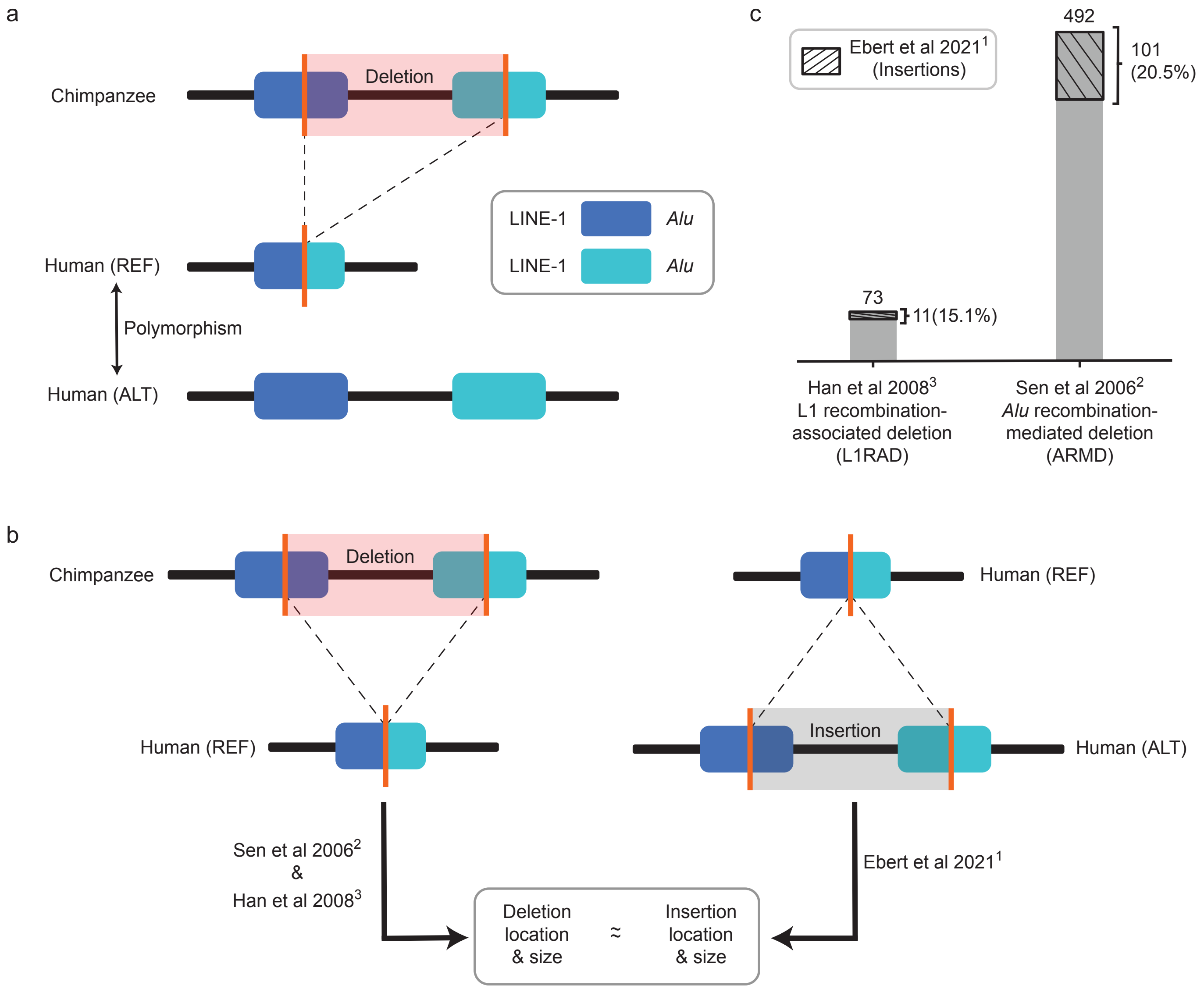

Supplementary Fig. 10. TEMR comparison between human and chimpanzee. a Schematic representing polymorphic TEMRs between human and chimpanzee genomes. **b** Schematic representing the method used to compare outputs between studies. **c** Comparing results between studies and identifying the percentage of TEMRs that are polymorphic within humans. REF, reference genome (GRCh38).

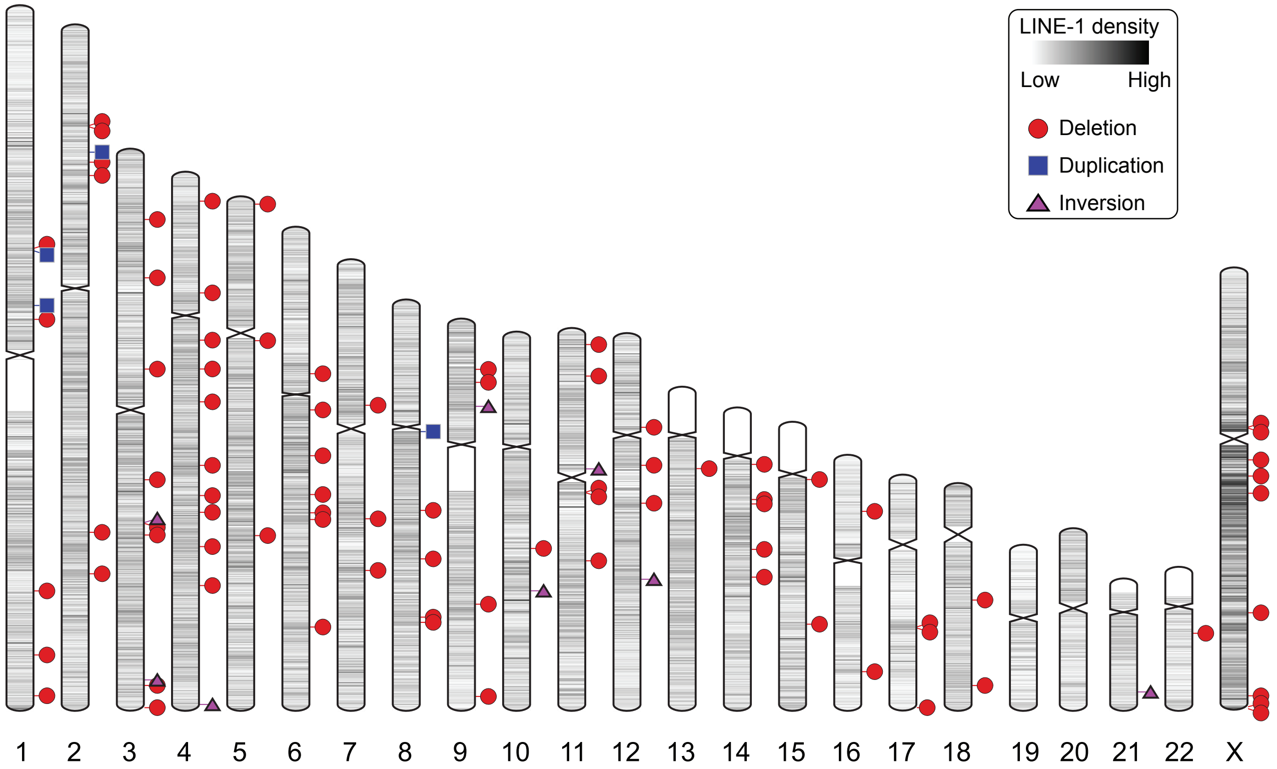

Supplementary Fig. 11. Ideogram displaying 96 LINE-1 TEMRs. The shapes represent the variant type (shape) and the shading represent the LINE-1 density.

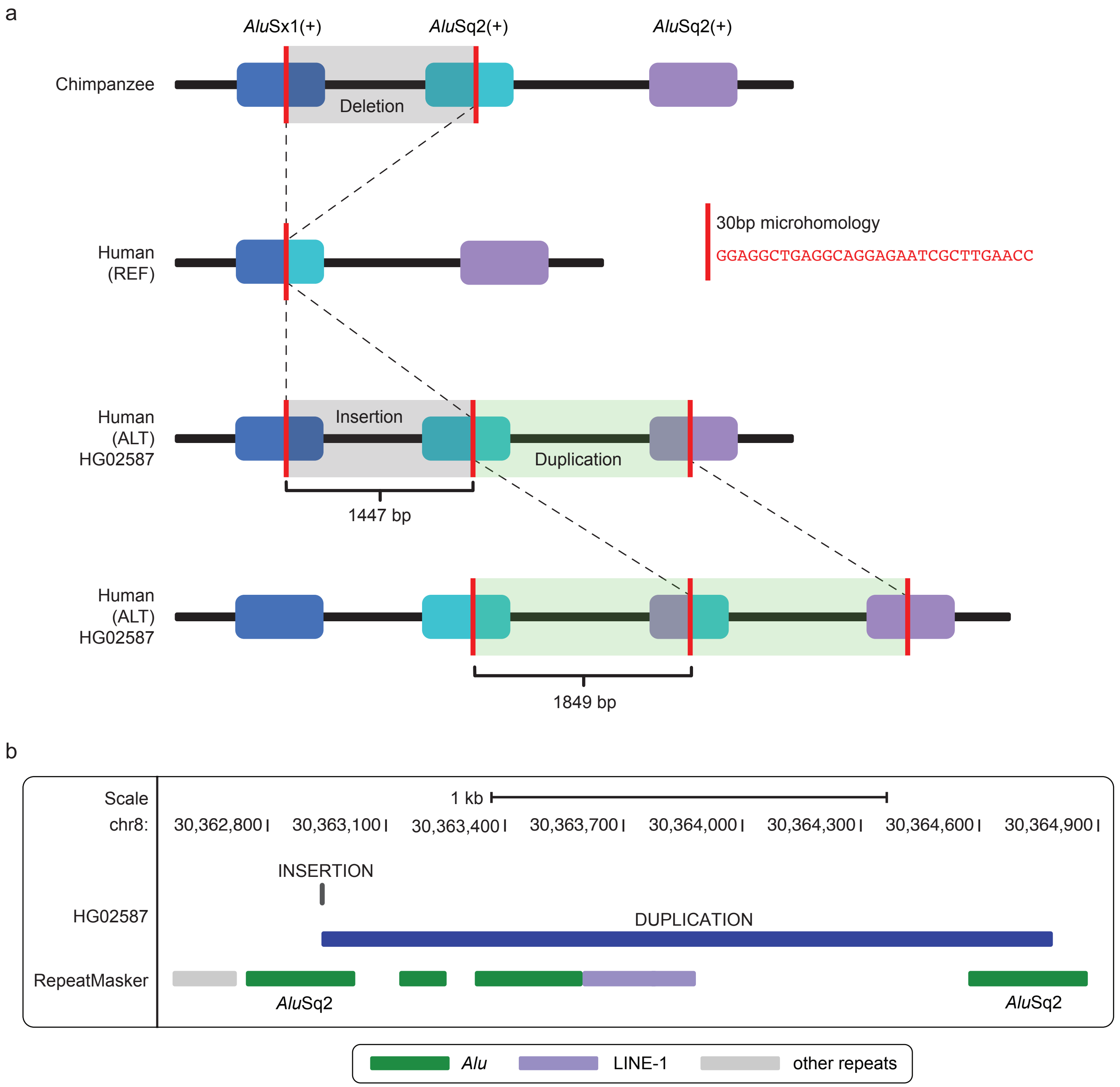

Supplementary Fig. 12. Example of a single *Alu* element involved in two separate rearrangements. a Polymorphic deletion between an *Alu*Sx1(blue) and an *Alu*Sq2(cyan) and a duplication between an *Alu*Sq2 (cyan) and an *Alu*Sq2 (purple) in HG02587. **b** UCSC genome browser depicting the insertion within an *Alu*Sq2 and duplication between an *Alu*Sq2 and an *Alu*Sq2.

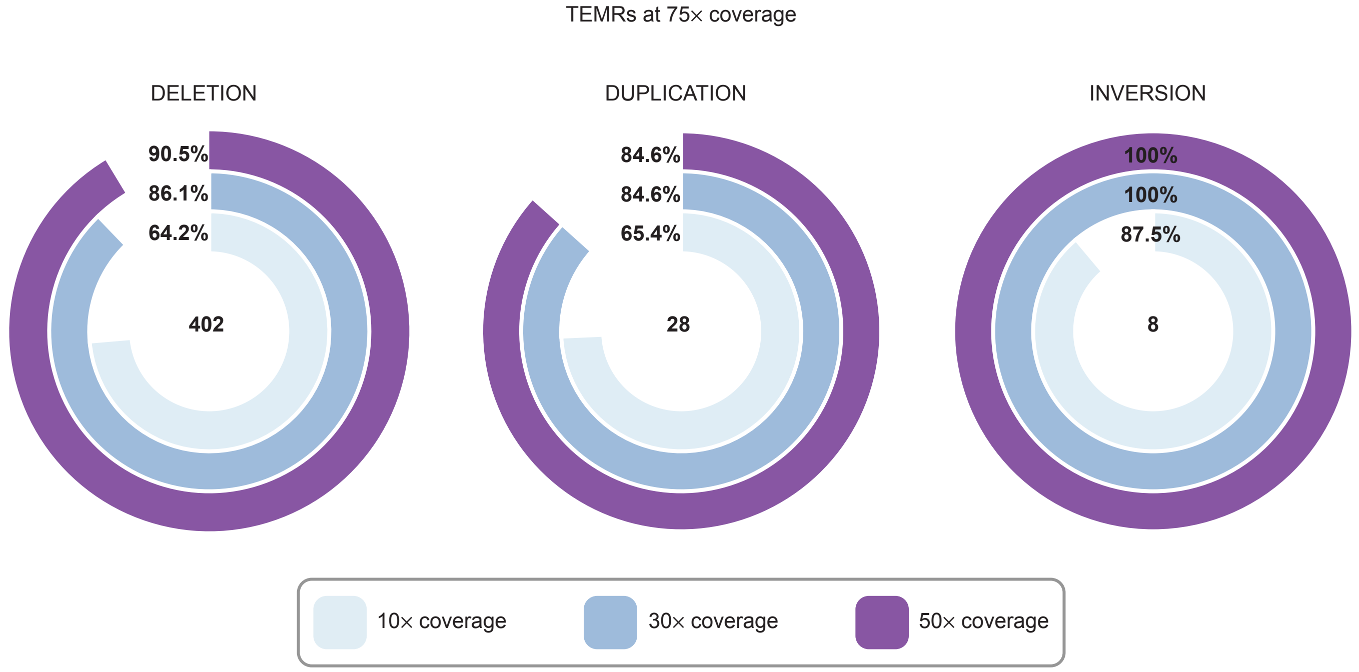

Supplementary Fig. 13. Downsampling analysis using short-read sequencing (SRS). Percentage of TEMRs identified using lower coverage (downsampled to 50×, 30× and 10×) SRS data compared to original (75×: 402 deletions, 28 duplications, and 8 inversions) SRS data used in this study.

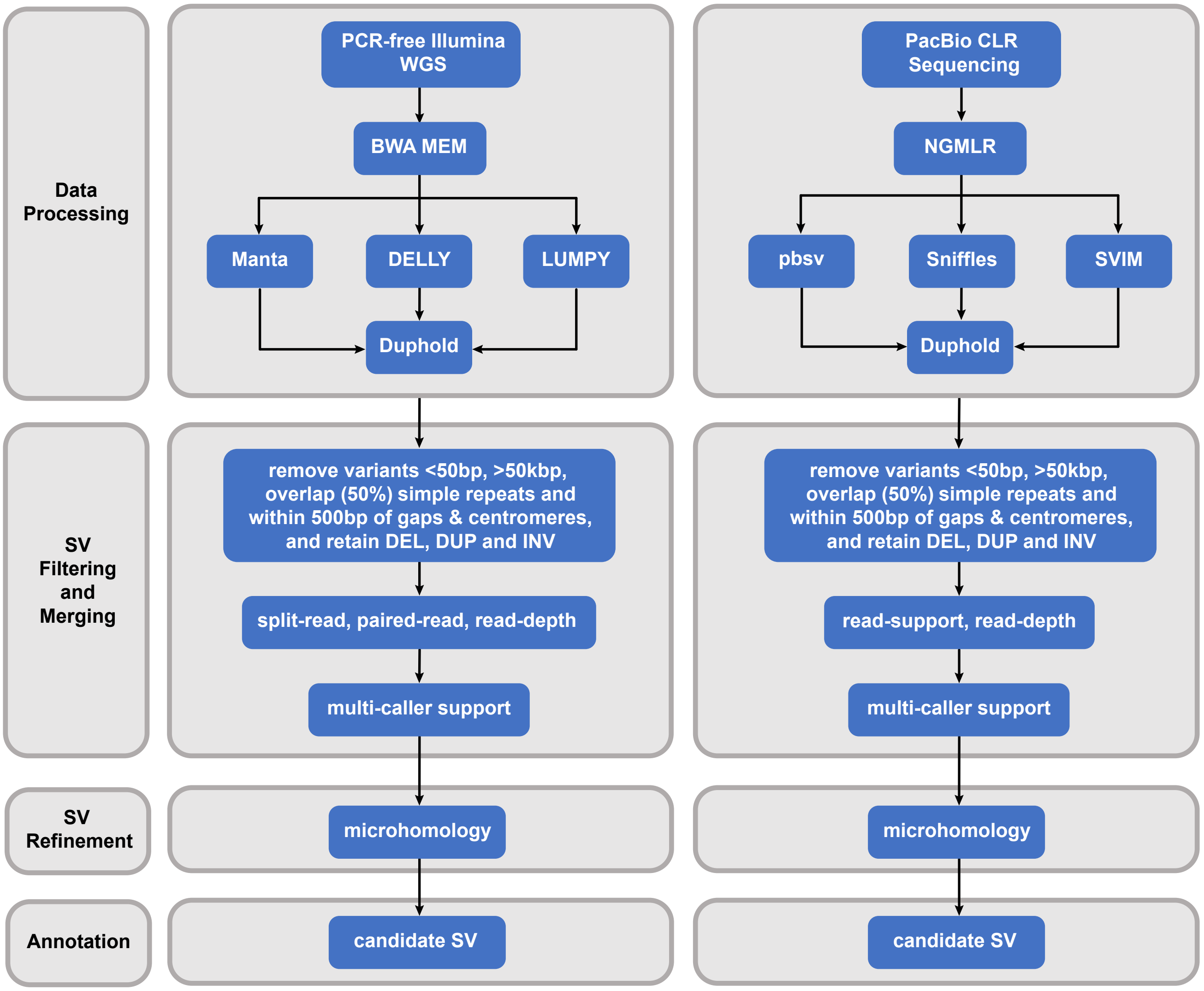

Supplementary Fig. 14. Pipeline for identifying non-redundant high-confident SVs using short-read sequencing and long-read sequencing data.

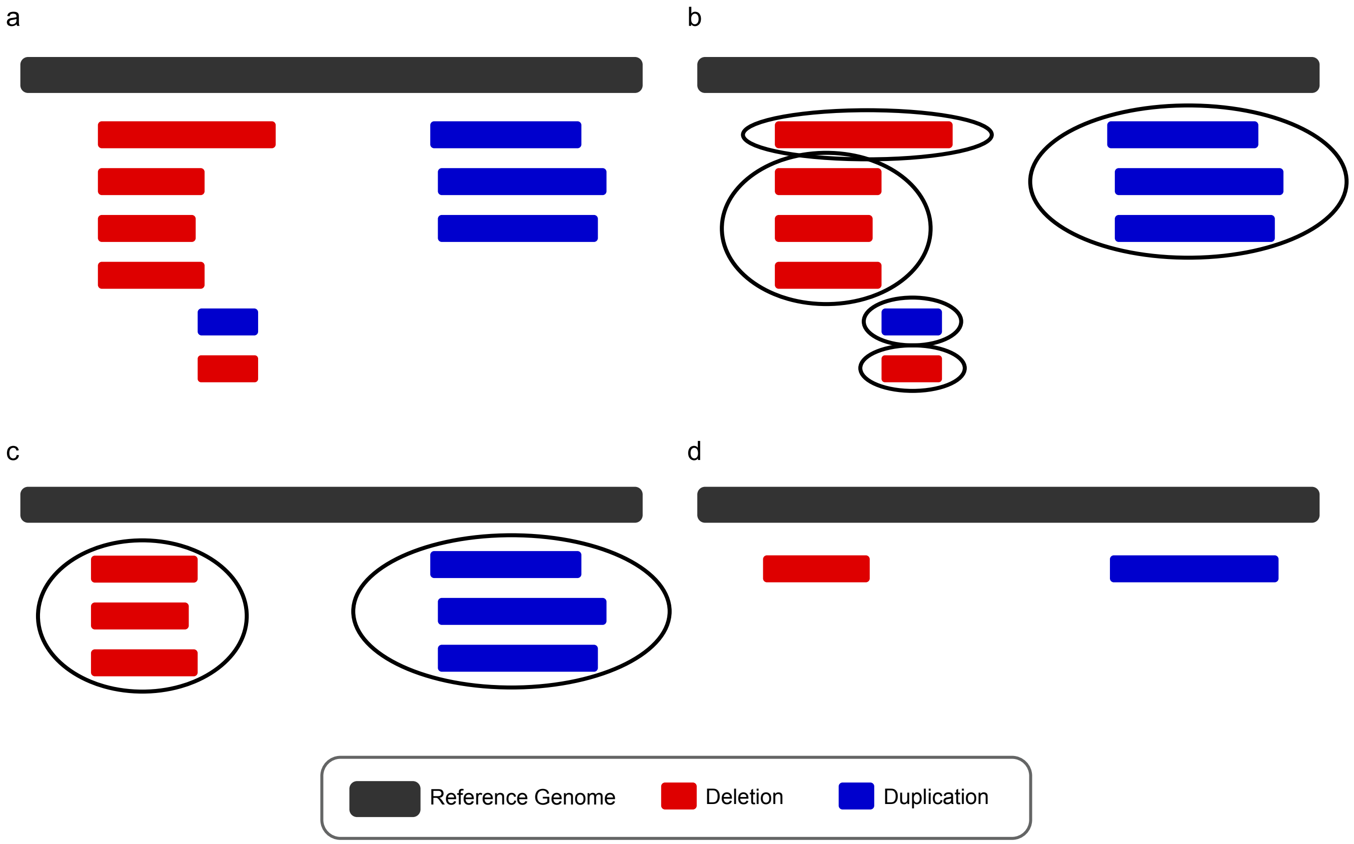

Supplementary Fig. 15. Rank-based merging SVs. a SVs are sorted based on coordinates. **b** SVs are clustered by 80% reciprocal overlap and type. **c** Clusters without SVs from at least 2 callers are removed. **d** SV with the highest rank within each cluster is retained and the rest are removed, resulting in one SV per cluster.

TE type	count	percentage	size in bp (min)	size in bp (median)	size in bp (max)
Alu	**397**	73.1	94	1163	24298
**L1**	**96**	17.7	93	4469	25423
ERVL-MaLR	14	2.6	121	1320.5	33050
ERV1	11	2	280	1831	35096
ERVL	8	1.5	57	356	2378
L2	6	1.1	219	935	11144
ERVK	3	0.6	5204	30201	43110
MIR	2	0.4	331	516.5	702
SVA	2	0.4	3260	6658.5	10057
TcMar-Mariner	2	0.4	301	547	793
TcMar-Tigger	1	0.2	169	169	169
hAT-Charlie	1	0.2	2001	2001	2001
Total	543				

Supplementary Table 1. Different type of homologous TEs involved in TEMRs.

CHR	POS	END	SV type	PCR forward primer	PCR reverse primer	Sanger forward primer	Sanger reverse primer	PCR & Sanger primer
chr1	16424661	16428623	DEL	GGATCCTCCCATCTCAGTATCTG	CCCTTGCAGCCTCATTTGTT	GGATCCTCCCATCTCAGTATCTG	CCCTTGCAGCCTCATTTGTT	Same
chr1	95076732	95077058	DEL	ATCAGCTGAGATCGGGAGTTT	GTTACTTGGGACACTGAGGTGA	ATCAGCTGAGATCGGGAGTTT	GTTACTTGGGACACTGAGGTGA	Same
chr1	206641337	206642314	DEL	CAGGTGGTGTTTGGGTTACATGA	GGATGGCTGGGTCAAATGGTATT	CAGGTGGTGTTTGGGTTACATGA	GGATGGCTGGGTCAAATGGTATT	Same
chr1	229237641	229238015	DEL	AGGGACTCTGCACATTCGG	GCAACCTGAAGTAACCTGATGT	AGGGACTCTGCACATTCGG	GCAACCTGAAGTAACCTGATGT	Same
chr1	247091230	247091618	DEL	AAATGGCAGGTGTGACAAGG	CAGCACTCTGTGAGTCTCTGTCT	AAATGGCAGGTGTGACAAGG	CAGCACTCTGTGAGTCTCTGTCT	Same
chr10	13675548	13676409	DEL	AATGGCAGGAAGATGGACCC	GCCTCTAACACCTGTCCGTT	AATGGCAGGAAGATGGACCC	GCCTCTAACACCTGTCCGTT	Same
chr11	677316	677770	DEL	GTCTCCTTATGTTGCCCAGG	CCACATGCAGCCAAGATTTCT	GTCTCCTTATGTTGCCCAGG	AGTGTCACTCTGTCGCCCA	Different
chr11	3961125	3961631	DEL	GAGAGCCTGGAGAGCAGTAG	ATACAGGTATTTGGCCGGGC	GAGAGCCTGGAGAGCAGTAG	ATACAGGTATTTGGCCGGGC	Same
chr11	5852754	5862263	DEL	AATGCACATTGTTACCTGGGT	GCTCTTGTGTTGTGCAAAGTT	AATGCACATTGTTACCTGGGT	GCTCTTGTGTTGTGCAAAGTT	Same
chr11	16974622	16975046	DEL	CCGATCAGCCAGCTTCCCTC	TGTGTCCTGATTGTGGTGGG	CCGATCAGCCAGCTTCCCTC	TGTGTCCTGATTGTGGTGGG	Same
chr11	65364535	65371883	DEL	AGTGCTTACTACTGGCCAGG	AGGTGGTGGAGGTTGAAGTG	AGTGCTTACTACTGGCCAGG	AGGTGGTGGAGGTTGAAGTG	Same
chr11	82145368	82152303	DEL	GCACGAAAGCCAGACATAGAT	TGACCTGTTAGACTAGCCTTAGG	GCACGAAAGCCAGACATAGAT	TGACCTGTTAGACTAGCCTTAGG	Same
chr12	1754845	1755160	DEL	GGAAGTGGCAGAGGGAGGATA	AGGAGGTTGAGATGGAGACC	AGTCTCGCTCTGTCACCCA	AGGAGGTTGAGATGGAGACC	Different
chr12	2843755	2844200	DEL	GCCCATAGTCCCAGCTACT	AGGTCCTGTATGATTTCCACCT	GCCCATAGTCCCAGCTACT	TCTCACTCTCACCCACGCT	Different
chr12	9028261	9029325	DEL	AATGGTGTGATCTCGGCTCA	AGCAGGCAATTCTAGGTGGA	AATGGTGTGATCTCGGCTCA	AGCAGGCAATTCTAGGTGGA	Same
chr12	15994723	15998587	DEL	GCGCGATCTCTGCTCACTG	GGGATGTAAGTTCAGCGGTCC	GCGCGATCTCTGCTCACTG	GGGATGTAAGTTCAGCGGTCC	Same
chr12	60018564	60019727	DEL	GGGATGCAAGGCTGGTTCAA	TCTGCTGTAGACACTCACAAAC	GGGATGCAAGGCTGGTTCAA	TCTGCTGTAGACACTCACAAAC	Same
chr12	64623682	64624699	DEL	TTCTTGCCAGTGTTCCTTTAGG	GCGCCTGTAGTTCCAGCTA	TTCTTGCCAGTGTTCCTTTAGG	GCGCCTGTAGTTCCAGCTA	Same
chr12	95616677	95618638	DEL	ACAGTCAGAACTGGAAGGGAAT	AGTAGATGTCAGAGGCCAGGTG	AGTCTCACTCTGTCGCCCA	AGTAGATGTCAGAGGCCAGGTG	Different
chr13	95368005	95372242	DEL	TGTGGATCATCAGGAGACCGT	CATGAGGCGGAAGTGTTGAGT	TGTGGATCATCAGGAGACCGT	CATGAGGCGGAAGTGTTGAGT	Same
chr14	34044819	34045627	DEL	TTCTGTCAGGGAGCTTTGCG	TGCCCGTAATCCCACCTACT	TTCTGTCAGGGAGCTTTGCG	TGCCCGTAATCCCACCTACT	Same
chr14	35136408	35145862	DEL	GGTGTGGGATGATGAGGGAA	TCAATTAAATGTACCACGGGCA	GGTGTGGGATGATGAGGGAA	TCAATTAAATGTACCACGGGCA	Same
chr14	63257033	63258189	DEL	CGGGTCATGAGGTCAGAAGT	TGGAACCTGTAGATGCTCAGC	CGGGTCATGAGGTCAGAAGT	AGTTTCACTCTTGTTGCCCAGG	Different
chr15	20376210	20384360	DEL	GCAGCCAACAGACACATGAA	ACCATGTGCTTCCCAGTCTG	GCAGCCAACAGACACATGAA	ACCATGTGCTTCCCAGTCTG	Same
chr16	4201466	4201845	DEL	CCAGTTCAAGTGATTCTCGTACC	CAGCCGACCTCCAACTTCTT	CCAGTTCAAGTGATTCTCGTACC	AAGGCGGGCAGATCATGAG	Different
chr16	19934228	19956257	DEL	GAGCACATGACCTCCCATTGG	GCTTCCAGGTCACAGGTAGGT	GAGCACATGACCTCCCATTGG	CTGCACCCATCAACCCATCAT	Different
chr16	76505236	76510128	DEL	ACCATATGACCCAGCAATCCC	ACAACACTGATGAACCTCCTTG	ACCATATGACCCAGCAATCCC	ACAACACTGATGAACCTCCTTG	Same
chr16	85115881	85116469	DEL	TCCGGGATTCAAGTGATTCTCC	GCAACTGTTCTCAAGTGGTTCA	TCCGGGATTCAAGTGATTCTCC	GCCTCATGCCTGTAATCCCAG	Different
chr17	820561	821700	DEL	ACACCTACTCAAGCGGCAGA	TGGTGAAGAGACAGGGAAGGG	AATTAGCTGGGCATGGTGGT	TGGTGAAGAGACAGGGAAGGG	Different
chr17	20707724	20711252	DEL	GGAGGAGGCTTGGCTAGAAA	TCAGGGAGCTGAGGTATGAGA	GGAGGAGGCTTGGCTAGAAA	TCAGGGAGCTGAGGTATGAGA	Same
chr17	35884154	35885055	DEL	CACCTCGCCCAGCTAGATTT	ATAGCCCATCAAGCCCAAAC	CACCTCGCCCAGCTAGATTT	ATAGCCCATCAAGCCCAAAC	Same
chr17	54081302	54090640	DEL	TCCTACTCCATCCCTCCCTTT	TGGTGTTTGAGTCATGAAGCCT	TCCTACTCCATCCCTCCCTTT	TGGTGTTTGAGTCATGAAGCCT	Same
chr18	47853242	47853436	DEL	GCGCAACTAGTCTGCTCAGTTC	TTCCCTCTCGAGACAGAGTCTT	GCGCAACTAGTCTGCTCAGTTC	AGTCTTGCTCTGTCGCCCA	Different
chr19	21453085	21455295	DEL	GTTTGTGGCATAATAACGCTTCA	GGGTTTCACTCGGTCTTCCA	GTTTGTGGCATAATAACGCTTCA	GGGTTTCACTCGGTCTTCCA	Same
chr19	49548637	49548743	DEL	TCTCCTGCCTCAGTCTTCCT	TCCTGCCCATGATTTCAGCA	GTTTCACCATGTTCGCCAGG	TCCTGCCCATGATTTCAGCA	Different
chr19	50051484	50058115	DEL	GCTGCGTCTATTTCCTGTGG	CTGCCATGTCCCTAGATCGT	GTCTCGCTCTGTTGCCCA	CTGCCATGTCCCTAGATCGT	Different
chr2	48624051	48630813	DEL	TGCGAACACTGGGTACTCC	TGAACCCAGCAGCTCATCAAA	TGCGAACACTGGGTACTCC	TGAACCCAGCAGCTCATCAAA	Same
chr2	172139520	172142752	DEL	GCCTCTAAGTGTTCCAGTGAAGG	AGGTGAAGGAAAGCAAGTTGGAG	GCCTCTAAGTGTTCCAGTGAAGG	CGAGGTGGGTGAACTCCTGA	Different
chr2	193824777	193834041	DEL	TGAGTTCAGTGGATGGCAGGA	AAAGCACTCCTCCGCAAATGT	TGAGTTCAGTGGATGGCAGGA	AAAGCACTCCTCCGCAAATGT	Same
chr2	227404174	227405319	DEL	ACAAAGATGAACACTGCTGCGA	GCTCCTCCATCCCTGTAAGCA	ACAAAGATGAACACTGCTGCGA	CCTCCAGGGTTCAAGCAATTCT	Different
chr21	31544310	31544418	DEL	AGGTGGGCAGATCGTTCAAG	GCAATGGCGTGATCTCAGCT	CACGCCTGTAATCCCAGCA	GCAATGGCGTGATCTCAGCT	Different
chr22	43984831	43986241	DEL	CGTTCCGTGTGTCATGTCGT	CCTCTGACGGATGTTGCATGT	CAAACTCCTGACCTCGTGATCC	CCTCTGACGGATGTTGCATGT	Different
chr3	9980228	9982164	DEL	TGGAGTCTGGAAAGTCAAGGTC	GGTATAGGCATACCCAGCGATA	TGGAGTCTGGAAAGTCAAGGTC	TGGAATCTCACTCTGTCTCCCA	Different
chr3	50305498	50310521	DEL	CCATCTGTCTGTCACCTGCC	GGGACACAAACACTGCGGAA	CCGGGTTCATGCCATTCTCC	GGGACACAAACACTGCGGAA	Different

chr3	125217451	125218319	DEL	CCAGGGAGGGATTGAGATAGTGA	ACTCAAGGTCAAGGTGAAGCAC	CCAGGGAGGGATTGAGATAGTGA	ACTCAAGGTCAAGGTGAAGCAC	Same
chr3	136302171	136307357	DEL	TGTAGCAACAGTGACCACCCA	CACGCCTGGCCTGTCATATTA	TGTAGCAACAGTGACCACCCA	GCAATATTCTTCCCCTCTCTTCTCC	Different
chr3	189374198	189382284	DEL	GCCCTAAGAGACAAGTACCGG	TCTGCGCTGGAGGATATGACA	GCCCTAAGAGACAAGTACCGG	TCTGCGCTGGAGGATATGACA	Same
chr3	195842345	195843061	DEL	TCTTTGCAAGCACTGGGAAGT	AGACGCCTGTTTCCCACCA	TCTTTGCAAGCACTGGGAAGT	CCACCATGCCCAGTCTGTTT	Different
chr4	56673800	56678541	DEL	ACCTCCACGACATCCTTCCA	CCACTGTGCCTAACCAACAATA	ACCTCCACGACATCCTTCCA	CGAGACCAGCTTGACCAACAT	Different
chr4	120165932	120166137	DEL	CAAATGCAATGAAGTAGTTGGCC	ATGCTCATGTCCTTTGTCCACT	CAAATGCAATGAAGTAGTTGGCC	ATGCTCATGTCCTTTGTCCACT	Same
chr5	2769309	2769526	DEL	ATGGAGTGATCTCGGCTTACC	AGCCTGTGGTATGCTACTGACC	ATGGAGTGATCTCGGCTTACC	AGCCTGTGGTATGCTACTGACC	Same
chr5	174703016	174705002	DEL	GTCTCACTCCCATTGCACAGG	GCCCAAGTAAACAGATCAATGCAC	GTCTCACTCCCATTGCACAGG	GCCCAAGTAAACAGATCAATGCAC	Same
chr6	103289589	103315014	DEL	TCAGAGGTCAAGAGGGTTGAA	TGGGCAAAGGATATGAACAGACAC	TCAGAGGTCAAGAGGGTTGAA	TGGGCAAAGGATATGAACAGACAC	Same
chr6	141227487	141228891	DEL	CTGGAATGATGAACAATGCCCA	CAACAATCAGGTGGCCAAATG	CTGGAATGATGAACAATGCCCA	CAACAATCAGGTGGCCAAATG	Same
chr7	51526615	51530992	DEL	ACTAGACCCACATCTCTCACCA	ACCATCTCACGCCAGTTAGAA	ACTAGACCCACATCTCTCACCA	ACCATCTCACGCCAGTTAGAA	Same
chr7	100729949	100743093	DEL	GACTGCCACACATAAATTTCTTTCT	GAGCAGAACCCTCTATGCTTGA	GACTGCCACACATAAATTTCTTTCT	GAGCAGAACCCTCTATGCTTGA	Same
chr7	109796632	109813844	DEL	CGTACCTGAAAGTGACGGC	ACCCAAAACCTCAGCTCTTTGT	CGTACCTGAAAGTGACGGC	ACCCAAAACCTCAGCTCTTTGT	Same
chr8	2921041	2921265	DEL	GGTGCGTGGATTAAGAGGTCA	AGGGTCTGGCTCTGTTGCT	GGTGCGTGGATTAAGAGGTCA	AGGGTCTGGCTCTGTTGCT	Same
chr8	74399197	74417845	DEL	TGATGTGCTGCTGGATTTGGT	GTCTCTCCACTTATCTAGGCCTTCT	TGATGTGCTGCTGGATTTGGT	GTCTCTCCACTTATCTAGGCCTTCT	Same
chr9	69189602	69190076	DEL	TGCTGAGTGTGGTGGCATAT	AAAGACAGTGAGGGAGGTGG	TGCTGAGTGTGGTGGCATAT	AAAGACAGTGAGGGAGGTGG	Same
chr9	111963571	111964556	DEL	CGTGAGCAGTGATCGTACCA	TGCCTCAGTTACCCAAGTAGC	CGTGAGCAGTGATCGTACCA	TGCCTCAGTTACCCAAGTAGC	Same
chr9	129264406	129265790	DEL	ACAAGCACCCATCACCACAC	GAGCAGCATAGTGAAAGCCCA	ACAAGCACCCATCACCACAC	TGGCACGCACCTGTAATCC	Different
chr9	133307392	133315932	DEL	TGTGCCGTGTGAGGTAGGAAA	GAAACCAACCAGAGTGCAGCA	TGTGCCGTGTGAGGTAGGAAA	TAGGGTACATGGGCACAATGT	Different
chrX	67903987	67910456	DEL	GTGAAGACCCAAGCCACAAAGA	GAAAGGCCAGCAGTCAAACCT	GTGAAGACCCAAGCCACAAAGA	GAAAGGCCAGCAGTCAAACCT	Same
chrX	73946227	73949977	DEL	ACCCATCCCTGATTGCATCTC	AAATATCTGCAGGCCGGGTG	AGTCTTGCTCTGTCACCCA	AAATATCTGCAGGCCGGGTG	Different
chrX	77865888	77868369	DEL	GTGGAGAGAAAGCAGACAGGG	CCACCTGCTCTGTCAATCATGA	GTGGAGAGAAAGCAGACAGGG	GTGGCTTGATCTCGGCTCAC	Different
chr12	123425279	123429398	DUP	AGTGATCCTCCCGCTTCAGG	AAGACACACAATTCGGCCAGG	AGTGATCCTCCCGCTTCAGG	AAGACACACAATTCGGCCAGG	Same
chr16	23454899	23455636	DUP	ATACAAGACTAGCCTGCCCAAC	CATGCTATGGATTGGAACAGTC	ATACAAGACTAGCCTGCCCAAC	CATGCTATGGATTGGAACAGTC	Same
chr20	43696486	43696990	DUP	GGGTTGAAGTGATTCTCCTGCC	CGGCAAATTCAGGAATGTTGGAG	GGGTTGAAGTGATTCTCCTGCC	CGGCAAATTCAGGAATGTTGGAG	Same
chr20	50816137	50816736	DUP	AGTTCTCACTCTGTCGCCCA	GGGAAATAATTAGCGGTTTGGCC	AGTTCTCACTCTGTCGCCCA	GGGAAATAATTAGCGGTTTGGCC	Same

Supplementary Table 2. Primers used for PCR and Sanger sequencing. Additional primers were designed for Sanger sequencing due to the presence of homopolymeric regions being present between the PCR primer and the breakpoint that could possibly cause premature polymerase slippage during sequencing.

All SVs (70 Total TEMRs)												
**SV caller**	**# TEMRs identified by each caller**	**# of TEMRs with 0 bp deviation**	**# of TEMRs with 1 bp deviation**	**# of TEMRs with 2+ bp deviation**		**# of TEMRs with 2+ bp deviation**	**min**	**q1**	**median**	**mean**	**q3**	**max**
delly	68	1	54	13		13	2	3	51	34.92	60	84
lumpy	62	9	3	50		50	2	6	35	34.32	49.8	89
manta	67	62	0	5		5	2	3	4	70.4	122	221
pav (HiFi)	70	63	3	4		4	4	4	13	17.5	26.5	40
pbsv (CLR)	64	47	3	14		14	2	3.25	12.5	22.93	34	90
sniffles (CLR)	70	3	6	61		61	2	6	12	33.82	33	354
svim (CLR)	67	4	4	59		59	2	4	12	29.27	37	358
**TEMRs with at least 1 bp junction homology (58 out of 70 Total TEMRs)**												
**SV caller**	**# SVs identified by each caller**	**# of SVs with 0 bp deviation**	**# of SVs with 1 bp deviation**	**# of SVs with 2+ bp deviation**		**# of TEMRs with 2+ bp deviation**	**min**	**q1**	**median**	**mean**	**q3**	**max**
delly	56	0	48	8		8	2	51.75	59.5	55	65.5	84
lumpy	50	0	2	48		48	2	6	36	35.44	50.3	89
manta	55	51	0	4		4	3	3.75	63	87.5	147	221
pav (HiFi)	58	57	0	1		1	40	40	40	40	40	40
pbsv (CLR)	53	42	1	10		10	2	4.25	21	28.7	40.8	90
sniffles (CLR)	58	0	3	55		55	2	6.5	12	36.45	35	354
svim (CLR)	56	2	3	51		51	2	4	19	32.65	39.5	358
**TEMRs with no junction homology (12 out of 70 Total TEMRs)**												
**SV caller**	**# TEMRs identified by each caller**	**# of TEMRs with 0 bp deviation**	**# of TEMRs with 1 bp deviation**	**# of TEMRs with 2+ bp deviation**		**# of TEMRs with 2+ bp deviation**	**min**	**q1**	**median**	**mean**	**q3**	**max**
delly	12	1	6	5		5	2	2	3	2.8	3	4
lumpy	12	9	1	2		2	2	4.75	7.5	7.5	10.3	13
manta	12	11	0	1		1	2	2	2	2	2	2
pav (HiFi)	12	6	3	3		3	4	4	4	10	13	22
pbsv (CLR)	11	5	2	4		4	2	3.5	4.5	8.5	9.5	23
sniffles (CLR)	12	3	3	6		6	2	5	5.5	9.67	8.25	31
svim (CLR)	11	2	1	8		8	3	4.75	5.5	7.75	9	21

Supplementary Table 3. Assessment of breakpoint accuracy between manual reconstruction and SV callers. A total of 70 TEMRs were validated using PCR and Sanger sequencing and their corresponding breakpoints were manually reconstructed. We compared the breakpoint accuracy of all seven callers used in this study and additional metrics were calculated for events with more than 2bp deviation from the manually reconstructed junctions.

Alu elements			LINE-1 elements	
*Alu*Y	*Alu*Sc		L1HS	L1M1
*Alu*Ya5	*Alu*Sc5		L1PA2	L1M2
*Alu*Yb8	*Alu*Sc8		L1PA3	L1M4a1
*Alu*Yc	*Alu*Sg		L1PA4	L1M4c
*Alu*Ye5	*Alu*Sg4		L1PA5	L1M5
*Alu*Yf1	*Alu*Sg7		L1PA6	L1MA1
*Alu*Yg6	*Alu*Sp		L1PA7	L1MA2
*Alu*Yh3	*Alu*Sq		L1PA8	L1MA3
*Alu*Yh3a3	*Alu*Sq2		L1PA11	L1MA4
*Alu*Yj4	*Alu*Sq4		L1PA13	L1MB5
*Alu*Yk2	*Alu*Sx		L1PA14	L1MB7
*Alu*Yk3	*Alu*Sx1		L1PA15	L1MB8
*Alu*Ym1	*Alu*Sx3		L1PA16	L1MC1
	*Alu*Sx4		L1PA17	L1MC4
	*Alu*Sz		L1PB1	L1MC4a
	*Alu*Sz6		L1PB2	L1MCa
	*Alu*Jb		L1PB3	L1MCc
	*Alu*Jo		L1PB4	L1MD
	*Alu*Jr		L1PBa	L1MD1
	*Alu*Jr4		L1PREC2	L1MDa
	Alu		L1P1	L1ME1
	FAM		L1P2	L1ME2
	FLAM_C		L1P4	L1ME2z
				L1ME3Cz
				L1ME3G
				L1ME4a
				L1MEc
				L1MEd
				L1MEf
				HAL1
				HAL1ME

Supplementary Table 4. List of 36 *Alu* and 54 LINE-1 elements involved in 493 TEMRs.

TE family	subfamily	Count (this study)	Percentage (this study)	Count (GRCh38)	Percentage (GRCh38)	p-value
Alu	*Alu*Y	195	24.6	139234	11.8	$2.341e^{-23}$
	*Alu*S	530	66.8	678131	57.4	$8.158e^{-8}$
	*Alu*J	64	8.1	309536	26.2	
	Others	5	0.6	54171	4.6	
(397 *Alu* TEMRs)	Total	794		1181072		
LINE-1	L1HS	4	2.1	1620	0.2	0.00034
	L1PA	103	53.6	121006	12.6	$2.946e^{-42}$
	L1M	60	31.3	747338	77.7	
	Others	25	13	92121	9.6	
(96 LINE-1 TEMRs)	Total	192		962085		

Supplementary Table 5. Count of *Alu* and LINE-1 subfamilies involved in 493 TEMRs. Two-tailed Fisher's exact *t*-test was used to calculate the p-value.

TE type	SV type	Mechanism	Percent similarity (median)
Alu	Deletion	HR	82.3
		NHE	80.6
	Duplication	HR	81.5
		NHE	79.5
	Inversion	HR	81.7
LINE-1	Deletion	HR	93.5
		NHE	47.9
	Duplication	HR	88.4
		NHE	60.2
	Inversion	HR	97.1

Supplementary Table 6. Median percent similarity between different TEs, SV types, and mechanisms.

Mechanism	TE	Orientation	Feature_1	Feature_2	r-value (spearman)	p-value (spearman)
HR	*Alu*	SAME	HOMLEN	PCT_SIMILARITY	0.1932	0.00026
			HOMLEN	SV_LENGTH	0.08868	0.09573
			PCT_SIMILARITY	SV_LENGTH	0.12577	0.01791
HR	LINE-1	SAME	HOMLEN	PCT_SIMILARITY	0.49028	0.0024
			HOMLEN	SV_LENGTH	-0.26838	0.1135
			PCT_SIMILARITY	SV_LENGTH	-0.24659	0.14711
NHE	*Alu*	SAME	HOMLEN	PCT_SIMILARITY	-0.34638	0.1143
			HOMLEN	SV_LENGTH	0.11569	0.60818
			PCT_SIMILARITY	SV_LENGTH	0.05251	0.81647
NHE	*Alu*	OPP	HOMLEN	PCT_SIMILARITY	0.23634	0.36111
			HOMLEN	SV_LENGTH	0.11883	0.64966
			PCT_SIMILARITY	SV_LENGTH	-0.2451	0.34305
NHE	LINE-1	SAME	HOMLEN	PCT_SIMILARITY	0.02663	0.88114
			HOMLEN	SV_LENGTH	0.13095	0.4604
			PCT_SIMILARITY	SV_LENGTH	0.29015	0.096
NHE	LINE-1	OPP	HOMLEN	PCT_SIMILARITY	0.02352	0.90922
			HOMLEN	SV_LENGTH	0.1013	0.62244
			PCT_SIMILARITY	SV_LENGTH	-0.32923	0.10052

Supplementary Table 7. Summary of the correlation analysis. Correlation analysis (two-tailed Spearman) within different categories based on mechanism (HR/NHE), TE type (*Alu*/LINE-1), and orientation (same/opposite). HOMLEN: homology length; PCT_SIMILARITY: percent similarity (calculated using swalign); SV_LENGTH: length of TEMR.

TEMRs overlapping					non-TEMRs overlapping (including polymorphic MEI)					non-TEMRs overlapping (excluding polymorphic MEI)			
**All genes**													
**Overlap status**	**RefSeq (curated + predicted)**	**RefSeq (curated only)**	**% of curated Refseq genes**		**Overlap status**	**RefSeq (curated + predicted)**	**RefSeq (curated only)**	**% of curated Refseq genes**		**Overlap status**	**RefSeq (curated + predicted)**	**RefSeq (curated only)**	**% of curated Refseq genes**
exonic	28	21	75		exonic	182	148	81.3		exonic	146	121	82.9
intronic	241	221	91.7		intronic	2244	1885	84		intronic	1021	860	84.2
proximal	52	40	76.9		proximal	314	226	72		proximal	169	132	78.1
**Total**	**321**	**282**	**87.9**		**Total**	**2740**	**2259**	**82.4**		**Total**	**1336**	**1113**	**83.3**
**Protein coding genes**													
**Overlap status**	**RefSeq (curated + predicted)**	**RefSeq (curated only)**	**% of curated Refseq genes**		**Overlap status**	**RefSeq (curated + predicted)**	**RefSeq (curated only)**	**% of curated Refseq genes**		**Overlap status**	**RefSeq (curated + predicted)**	**RefSeq (curated only)**	**% of curated Refseq genes**
exonic	20	19	95		exonic	119	112	94.1		exonic	95	89	93.7
intronic	212	204	96.2		intronic	1690	1632	96.6		intronic	775	747	96.4
proximal	37	33	89.2		proximal	171	165	96.5		proximal	96	92	95.8
**Total**	**269**	**256**	**95.2**		**Total**	**1980**	**1909**	**96.4**		**Total**	**966**	**928**	**96.1**

Supplementary Table 8. Total count of Exonic, intronic and proximal SVs. We used the RefSeq dataset from NCBI and calculated the percentage of TEMRs overlapping only the curated gene set.

Category	Name	Version	Settings used	URL
Illumina WGS	BWA-MEM	0.7.17	default	https://github.com/lh3/bwa
	Manta	1.3.2	high sensitivity calling	https://github.com/Illumina/manta
	LUMPY	0.2.13	default	https://github.com/arq5x/lumpy-sv
	DELLY	0.7.8	default	https://github.com/dellytools/delly
	Duphold	0.2.1	default	https://github.com/brentp/duphold
	mosdepth	0.3.2	default	https://github.com/brentp/mosdepth
PacBio WGS	pbh5tools	0.8.0	default	https://github.com/PacificBiosciences/pbh5tools
	NGMLR	0.2.6	default	https://github.com/philres/ngmlr
	Sniffles	1.0.7	default with -s 5	https://github.com/fritzsedlazeck/Sniffles
	pbsv	2.2.0	default	https://github.com/pacificbiosciences/pbsv/
	SVIM	1.4.0	default	https://github.com/eldariont/svim
	pav	1.1.0	default	https://github.com/EichlerLab/pav
General	BioConda			https://bioconda.github.io/index.html
	samtools	1.7		https://github.com/samtools/samtools
	bcftools	1.9		https://github.com/samtools/bcftools
	bedtools	2.27.1		https://github.com/arq5x/bedtools2
	swalign	0.3.4		https://github.com/mbreese/swalign
	Ensembl Variant Effect Predictor	release 106	Refseq transcripts database	https://useast.ensembl.org/info/docs/tools/vep/index.html
Raw data	1000GP			https://www.internationalgenome.org/data
Python packages	pandas	1.0.5		https://github.com/pandas-dev/pandas
	scipy	1.5.0		https://github.com/scipy/scipy
	numpy	1.19.1		https://github.com/numpy/numpy
	biopython	1.78		https://github.com/biopython/biopython
Plots (python)	matplotlib	3.2.2		https://github.com/matplotlib/matplotlib
	seaborn	0.11.0		https://github.com/mwaskom/seaborn
	PyWaffle	0.6.1		https://github.com/gyli/PyWaffle
	matplotlib-venn	0.11.5		https://github.com/konstantint/matplotlib-venn
	brokenaxes	0.4.2		https://github.com/bendichter/brokenaxes
	upsetplot	0.6.0		https://github.com/jnothman/UpSetPlot
Plots (R)	RIdeogram	0.2.2		https://cran.r-project.org/web/packages/RIdeogram/vignettes/RIdeogram.html

Supplementary Table 9. Tools and packages.

TEMR	Copy Number status	Restriction enzyme	Forward Primer	Probe	Reverse Primer
chr17:43359210-43365251	Gain	AluI	AGTCCTCTAGATGGGAGGTG	TCACATGTACATCGGCCTGGTGT	TGAGGAACAGTTTCTGGTGG
chr17:43359210-43365251	Loss	AluI	CGTGTAAACGCAACACACAA	CGGTCCCAACTGATGCAAATGGC	GTCGCTAACGGGAGATTGTC
chr16:81765053-81765596	Gain	HaeIII	TTGGACACAGAGACATGCAC	GCAACACAGCGACACACAGGG	TCAGTTGTCACATGGTGCTC
chr16:3632625-3634268	Gain	AluI	TCTTTGCCCTTGAGTTTGGT	TCCACTTTCACCTGATGCTATACCACTT	GAGGGAACTTGTACAACCCA
chr14:21581819-21590876	Gain	AluI	CCTGACCTCAGGTAGTCTGT	GGCCTCCCAAAGTGCTGGGA	GCATTAAAGACAAGGCAGGC
**Reference gene**					
RPP30	Neutral	None	CCCACTGAGAGATGTTTAGTAAG	ACAGTGAGGTAGTTCCATGAATCATGCT	ACAGAACACTCACATCCAAA

Supplementary Table 10. Restriction enzymes, primers and probes used for ddPCR.

Supplementary References

1. Ebert, P. *et al.* Haplotype-resolved diverse human genomes and integrated analysis of structural variation. *Science* **372**(2021).
2. Sen, S.K. *et al.* Human genomic deletions mediated by recombination between Alu elements. *Am J Hum Genet* **79**, 41-53 (2006).
3. Han, K. *et al.* L1 recombination-associated deletions generate human genomic variation. *Proc Natl Acad Sci U S A* **105**, 19366-71 (2008).
