# Supplementary material for: Transposable element-mediated rearrangements are prevalent in human genomes
Source: Nat Commun. 2022 Nov 19;13:7115. doi: 10.1038/s41467-022-34810-8 (PMC9675761; doi:10.1038/s41467-022-34810-8)
Supplement: Supplementary file 3 — Description of Additional Supplementary Files [file 41467_2022_34810_MOESM3_ESM.pdf]

Supplementary Data 1

List of 445 TE-mediated deletions

Supplementary Data 2

List of 33 TE-mediated duplications

Supplementary Data 3

List of 15 TE-mediated inversions
