# Supplementary material for: Transposable element-mediated rearrangements are prevalent in human genomes
Source: Nat Commun. 2022 Nov 19;13:7115. doi: 10.1038/s41467-022-34810-8 (PMC9675761; doi:10.1038/s41467-022-34810-8)
Supplement: Supplementary file 5 — Reporting Summary [file 41467_2022_34810_MOESM5_ESM.pdf]

Corresponding author(s): Christine R Beck

Last updated by author(s): Nov 3, 2022

Reporting Summary

Nature Portfolio wishes to improve the reproducibility of the work that we publish. This form provides structure for consistency and transparency in reporting. For further information on Nature Portfolio policies, see our Editorial Policies and the Editorial Policy Checklist.

Statistics

For all statistical analyses, confirm that the following items are present in the figure legend, table legend, main text, or Methods section.

n/a	Confirmed	
☐	☒	The exact sample size (*n*) for each experimental group/condition, given as a discrete number and unit of measurement
☐	☒	A statement on whether measurements were taken from distinct samples or whether the same sample was measured repeatedly
☐	☒	The statistical test(s) used AND whether they are one- or two-sided<br>*Only common tests should be described solely by name; describe more complex techniques in the Methods section.*
☒	☐	A description of all covariates tested
☒	☐	A description of any assumptions or corrections, such as tests of normality and adjustment for multiple comparisons
☐	☒	A full description of the statistical parameters including central tendency (e.g. means) or other basic estimates (e.g. regression coefficient) AND variation (e.g. standard deviation) or associated estimates of uncertainty (e.g. confidence intervals)
☐	☒	For null hypothesis testing, the test statistic (e.g. *F*, *t*, *r*) with confidence intervals, effect sizes, degrees of freedom and *P* value noted<br>*Give P values as exact values whenever suitable.*
☒	☐	For Bayesian analysis, information on the choice of priors and Markov chain Monte Carlo settings
☒	☐	For hierarchical and complex designs, identification of the appropriate level for tests and full reporting of outcomes
☒	☐	Estimates of effect sizes (e.g. Cohen's *d*, Pearson's *r*), indicating how they were calculated

Our web collection on statistics for biologists contains articles on many of the points above.

Software and code

Policy information about availability of computer code

Data collection	No new sequencing data was generated in this study.
Data analysis	Tools and packages used for analysis in this study<br>short-read sequencing: BWA-MEM(0.7.17), Manta(1.3.2),LUMPY(0.2.13), DELLY(0.7.8), Duphold(0.2.1), mosdepth(0.3.2)<br>long-read sequencing: pbh5tools(0.8.0), NGMLR(0.2.6), Sniffles(1.0.7), pbsv(2.2.0), SVIM(1.4.0), pav(1.1.0)<br>Python packages: pandas(1.0.5), scipy(1.5.0), numpy(1.19.1), biopython(1.78), matplotlib(3.2.2), seaborn(0.11.0), PyWaffle(0.6.1),matplotlib-venn(0.11.5), brokenaxes(0,4,2), upsetplot(0.6.0)<br>R packages: RIdeogram(0.2.2)<br>Others: samtools(1.7), bcftools 1.9), bedtools (2.27.1), swalign(0.3.4), Ensembl Variant Effect Predictor(release 106)

For manuscripts utilizing custom algorithms or software that are central to the research but not yet described in published literature, software must be made available to editors and reviewers. We strongly encourage code deposition in a community repository (e.g. GitHub). See the Nature Portfolio guidelines for submitting code & software for further information.

Data

Policy information about availability of data

All manuscripts must include a data availability statement. This statement should provide the following information, where applicable:

- Accession codes, unique identifiers, or web links for publicly available datasets
- A description of any restrictions on data availability
- For clinical datasets or third party data, please ensure that the statement adheres to our policy

All raw sequencing data used in this study were downloaded from publicly available databases.
1. All sequencing data used in this study were directly downloaded from publicly available database at International Genome Sample Resource (IGSR) at https://www.internationalgenome.org/data-portal/data-collection/structural-variation (Chaisson et al. 2019); https://www.internationalgenome.org/data-portal/data-collection/hgsvc2 (Ebert et al. 2021).
2. The human reference genome GRCh38/hg38 was downloaded from UCSC Genome Browser at https://hgdownload.soe.ucsc.edu/goldenPath/hg38/chromosomes/.
3. NCBI Refseq dataset was downloaded from https://www.ncbi.nlm.nih.gov/projects/genome/guide/human/index.shtml.
4. L1 recombination associated deletion (Han et al. 2008)data was download from https://biosci-batzerlab.biology.lsu.edu/supplementary_data/Han_et_al_L1RAD_SI_Table_S4.doc.
5. Alu recombination-mediation deletion (Sen et al. 2006) data was downloaded from https://biosci-batzerlab.biology.lsu.edu/supplementary_data/Sen_et_al_Suppl_Data.zip.
6. SV and TEMR files have been deposited in Zenodo with the following accession code: 10.5281/zenodo.7272154. Pipeline used in this study can be found at https://github.com/parithi-b/TEMR_analysis_pipeline
7. Primers used for PCR, Sanger and ddPCR experiments can be found in the supplementary table.
8. All other data used for analysis and figures are available within supplementary information and supplementary data.

Human research participants

Policy information about studies involving human research participants and Sex and Gender in Research.

Reporting on sex and gender	N/A
Population characteristics	N/A
Recruitment	N/A
Ethics oversight	N/A

Note that full information on the approval of the study protocol must also be provided in the manuscript.

Field-specific reporting

Please select the one below that is the best fit for your research. If you are not sure, read the appropriate sections before making your selection.

☒ Life sciences ☐ Behavioural & social sciences ☐ Ecological, evolutionary & environmental sciences

For a reference copy of the document with all sections, see nature.com/documents/nr-reporting-summary-flat.pdf

Life sciences study design

All studies must disclose on these points even when the disclosure is negative.

Sample size	Three well-characterized genomes representative of: (1) population admixture, Puerto Rican HG00733 (PUR); (2) low diversity, Southern Han Chinese HG00514 (CHS); and (3) high diversity, Yoruban NA19240 (YRI) were used in this study. Additionally, these three individuals have been studies as a part of the 1000GP phase 3, HGSVC phase 1, and HGSVC phase 2 studies, which provides us with extensive genomic data ranging from short-read sequencing to long-read sequencing, Bionano genomics data and RNA-Seq that can be used for further understanding of TEMRs.
Data exclusions	No data were excluded.
Replication	All sequencing data can be downloaded from IGSR. The sequence analyses can be replicated using the analysis flow that has been posted on github.
Randomization	Samples were randomly selected from the population.
Blinding	All samples were analyzed by the same pipeline.

Reporting for specific materials, systems and methods

We require information from authors about some types of materials, experimental systems and methods used in many studies. Here, indicate whether each material, system or method listed is relevant to your study. If you are not sure if a list item applies to your research, read the appropriate section before selecting a response.

Materials & experimental systems

n/a	Involved in the study
☒	☐ Antibodies
☐	☒ Eukaryotic cell lines
☒	☐ Palaeontology and archaeology
☒	☐ Animals and other organisms
☒	☐ Clinical data
☒	☐ Dual use research of concern

Methods

n/a	Involved in the study
☒	☐ ChIP-seq
☒	☐ Flow cytometry
☒	☐ MRI-based neuroimaging

Eukaryotic cell lines

Policy information about cell lines and Sex and Gender in Research

Cell line source(s)	All cell lines (HG00512, HG00513, HG00514, HG00731, HG00732, HG00733, NA19239, NA19238, NA19240) used for PCR, Sanger and ddPCR experiments in this study can be obtained from Coriell Biorepository.
Authentication	SVs obtained from the sequencing data and validated by Sanger sequencing were compared with previously published data and used to authenticate each cell line.
Mycoplasma contamination	All cell lines used in this study tested negative for mycoplasm presence
Commonly misidentified lines (See ICLAC register)	N/A
